# An Experimental Investigation of Controlled Changes in Wettability of Laser-Treated Surfaces after Various Post Treatment Methods

**DOI:** 10.3390/ma14092228

**Published:** 2021-04-26

**Authors:** Tomáš Primus, Pavel Zeman, Jan Brajer, Pavel Kožmín, Šimon Syrovátka

**Affiliations:** 1 Department of Production Machines and Equipment, Faculty of Mechanical Engineering, Czech Technical University in Prague, 166 07 Prague, Czech Republic; P.Zeman@rcmt.cvut.cz (P.Z.); J.Brajer@rcmt.cvut.cz (J.B.); 2 Hofmeister s. r. o., 301 00 Plzeň, Czech Republic; kozmin@hofmeister.cz (P.K.); syrovatka@hofmeister.cz (Š.S.)

**Keywords:** laser, wettability, post-processing, surface, Ti6Al4V alloy

## Abstract

In this paper, a quick nanosecond laser micro structuring process was employed to change the surface wettability of Ti6Al4V alloy. The same laser structuring method was used throughout, but with varying input fluence. The laser processing parameters resulted in high surface melting. After laser treatment, four post-processing methods were used, namely high vacuum, low temperature annealing, storage in a polyethylene bag, and storage in ambient air. Subsequently, the water droplet contact angle was measured over a long time period of 55 days. The results show that the sample stored in ambient air remained hydrophilic. On the other hand, the sample post-processed in a vacuum chamber behaved hydrophobically with a contact angle of approximately 150°. Other post-processing did not lead to specific wettability behavior. After wettability testing, all samples were cleaned ultrasonically in distilled water. This cleaning process led to annulation of all obtained properties through post-processing. In summary, this paper shows that it is more important to study surface chemistry than topography in terms of effects on wettability. Moreover, surface wettability can be controlled by laser structuring, post-processing, and surface cleaning.

## 1. Introduction

Many methods can be used to change surface properties, particularly mechanical and chemical methods, as well as physical methods [1]. All of these methods lead to changes in surface roughness and increase or decrease the free surface area, and some of them lead to changes in the surface chemistry [2]. A special method for changing the afore-listed surface properties is surface structuring. The goal of surface structuring is to improve corrosion resistance, wear resistance, biocompatibility, frictional properties, anti-icing properties, self-cleaning properties etc. [3,4]. Today, many scientists studying surface modifications begin with a surface wettability understanding which can predict surface properties [3,5]. On the one hand, hydrophilic surfaces with a contact angle of less than 90 degrees should improve biocompatibility and cell growth as well as frictional properties [4,6,7,8]. On the other hand, hydrophobic and superhydrophobic surfaces with a contact angle of more than 90 degrees, or 150 degrees, can reduce bacterial adhesion [9,10], improve corrosion and wear resistance [11], anti-icing, or antibiofouling behavior [12], and lower the coefficient of friction (CoF) [13]. The application of surfaces with low CoF can be found in automotive, e.g., in a piston/cylinder system [14]. In cutting tools, lowering friction on a rake face by laser produced dimples leads to a decrease of cutting forces and prolongs tool life [15,16]. Anti-icing properties could be applied on aircraft wings, air power plants, or ships [17].

Surface wettability properties are dependent on topography and chemistry [5,6]. In line with this knowledge, laser machining seems to be a suitable tool for the structuring of surfaces [18,19]. Laser surface texturing allows for the fabrication a lot of various structures, such as simple lines, dots, grids, or more complex structures, finding inspiration in nature, such as lotus leaf, shark skin, butterfly wings, etc. [20]. Lasers can also produce hierarchical structures with many perspectives [21], especially for super-hydrophobic surfaces fabricated by combination of nanosecond and femtosecond laser surface texturing, as reported by [22].

According to [23], a hydrophobic surface can only be achieved through exposure to ambient air. Samples taken after laser processing tend to be hydrophilic with time-dependent growth into hydrophobicity. Basically, the samples are either hydrophilic or hydrophobic over time. During the thermal laser process, the surface roughness is changed. A change of chemical properties takes place through the diffusion of oxides into the melt pool [24]. Other chemical changes may occur during further processing. Jagdheesh et al. [25] studied how vacuum post-processing of laser treated samples affected formation of superhydrophobic properties. Another research group led by Chi-Vinh Ngo [26] studied an additional low-temperature annealing post-process at 100 °C designed to achieve superhydrophobic behavior. Moreover, they used a nanosecond pulsed laser treatment to prepare the original structures. Huerta-Murillo et al. [22] applied a combination of nanosecond and femtosecond laser treatment with post-processing storage in polyethylene bags to prepare a superhydrophobic surface.

As already mentioned, hydrophilic and hydrophobic surfaces have potential application in medicine, especially in implantology. After implant implantation, proteins arrive first and the process of healing begins. Thus, protein adsorption to the implant surface is crucial for a proper healing process. According to [27], proteins adhere better to hydrophilic surfaces than to hydrophobic surfaces. As a result, using laser modification to change physical and chemical surface properties, e.g., a thicker oxide layer, the micro and nano structure, promotes better bone binding with the implant. Before biomaterial becomes an implant, it must first be tested in vitro, in vivo on animals and then clinically tested on patients. Implants must be sterilized before each test. There are many types of sterilization, such as plasma or UV, but the most common is steam sterilization at a high temperature [28]. The aim of this process is to remove all bacteria from the surface. However, this process can remove the effect of hydrophobicity from the surface [28,29].

In summary, researchers have shown that laser and post-processing, namely high vacuum, low-temperature annealing, and storage in polyethylene bags, can be suitable tools for preparing time-stable hydrophobic or hydrophilic surfaces. However, the stability of hydrophobicity remains an unresolved issue. In this paper, we present the effects of ultrasonic cleaning on hydrophobic surfaces and the process of quick transition between hydrophilic and hydrophobic states on titanium grade 5 sample. Titanium grade 5, also known as Ti6Al4V, is the most used titanium alloy, finding automotive, aeronautical, and medical applications [4]. Advantages of Ti6Al4V include high biocompatibility and a modulus of elasticity almost similar to human bone. It is important to investigate the change in the wettability of this alloy in reference to these properties [29,30]. Moreover, Ti6Al4V can be easily machined by laser due to its low thermal conductivity and standard ablation behavior [31].

In addition, we used laser structuring to prepare samples with the same topography, which can be hydrophilic or hydrophobic according to post-process conditions. This post-process helped us conserve sample properties over time. Using laser micromachining and post-processing, we showed that is possible to prepare one structure with controlled wettability. This research can lead to applications in various branches of industry. There is potential in the production of medical surfaces with antibacterial hydrophobic properties as well as areas that are hydrophilic and could lead to better cell adhesion [32,33].

## 2. Materials and Methods

### 2.1. Materials

Four cylindrical samples (diameter of 60 mm, height of about 20 mm) made of Ti6Al4V alloy were used for laser surface patterning. The surface chemical composition is specified in Table 1 The initial surface roughness after a turning process was Ra = 0.32 ± 0.2 μm and Rz = 1.6 ± 0.1 μm for all rollers. The rollers were marked as sample numbers 1 through 4. After the laser process, the four post-process conditions were applied, namely high vacuum, ambient air, low temperature annealing, and polyethylene bags. The titanium alloy grade 5 (Ti6Al4V) used in this experiment is now the most widely used implant material.

### 2.2. Laser System and Processing Parameters

**Figure 1:**
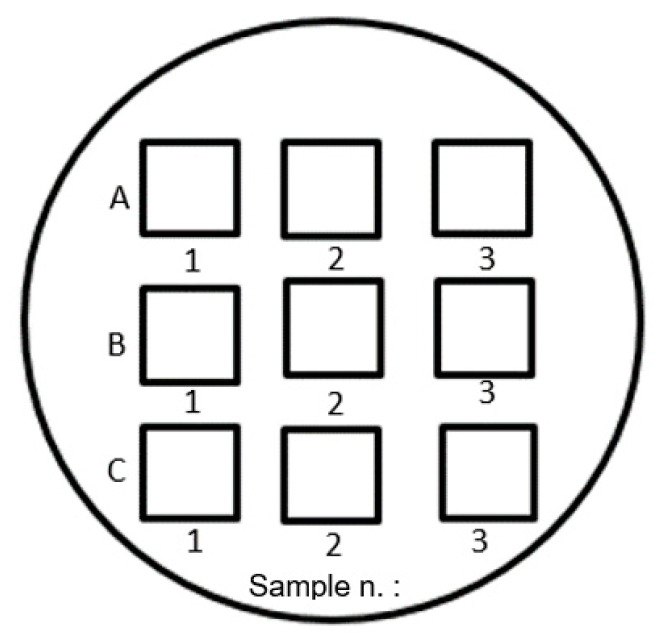
Distribution and indication of laser patterned samples.

A near-infrared laser source was used to create patterns on the samples. The pulsed laser source was a Nd:YAG with a wavelength of 1064 nm and pulse duration of 120 nanoseconds. The pulses had a non-polarized Gaussian profile with an average output power of 50 W. The laser beam was focused on the rollers using an F-Theta lens with a focusing distance of 132 mm. The focused beam had a spot size of 0.15 mm. The beam movement was provided by a galvo head with a maximum speed of 2000 mm/s. The maximum repetition rate was 50 kHz. The laser patterned samples were prepared in atmospheric conditions with nine areas of 12 × 12 mm^2^. A total of three different patterns, each in three repetitions, were prepared on four rollers. The laser patterns were indicated as A, B, C and repetitions in rows were indicated as 1, 2, 3. The distribution and marking of all patterns can be seen in Figure 1. All patterns were fabricated using the same path strategy but with varying laser energy input.

Inspired by dental implants, the sandblasted-like structures were created by a ns laser. A line-like pattern was set to obtain this type of structure. The hatch distance was set to 0.05 mm. After patterning the entire area, the laser paths were rotated 45 degrees and the process was repeated. A total of four rotations were used to prepare each structure. For the structure indicated as C, the patterning process was repeated four times. The laser parameters are shown in Table 2.

After the laser process, the samples were exposed to various environments. Sample no. 1 was stored under high vacuum conditions for 16 h. A turbomolecular pump with up to 8·10^−7^ Pa was used to create the low-pressure area in the chamber. Sample no. 2 was annealed at a low temperature (100 °C) in an oven. Sample no. 3 was stored in ambient air at 21 °C and normal humidity (50%) and pressure (1003.7 hPa). Sample no. 4 was stored in a polyethylene (C_2_H_4_) bag during the entire test period. Table 3 summarizes the samples and types of post-processing.

### 2.3. Surface Characterization

Surface properties were characterized in order to study surface morphology and chemistry. An optical 3D surface measurement system by Alicona Imagining GmbH (InfiniteFocus G5) was employed to analyze the depth, roughness and profile of the laser fabricated patterns. Laser confocal microscopy Keyence VK-X1000 (KEYENCE CORPORATION, Osaka, Japan) was used for measuring the specific parameters of surface roughness.

The changes in the wetting characteristics of laser treated and post-process samples were analyzed through measurement of the static contact angle using the sessile drop method with a video-based static contact angle computing device (OCA 15 from Data Physics Instruments). Droplets of distilled deionized water were applied in a volume of 8 μL. The contact angle values are the averages of three measurements. The total measurement time was 55 days. After this period, the samples were cleaned ultrasonically for 15 min in deionized water. Then, the samples were dried, and the static contact angles were measured again.

A Zeiss field-emission scanning electron microscope (FESEM; ULTRA PLUS, Jena, Germany) equipped with an energy-dispersive spectrometer from Oxford Instruments (EDS; X-Max 50) was used to detect changes in surface chemistry. In addition, Raman spectroscopy was employed to identify the form of titanium oxide.

## 3. Results

### 3.1. Laser Patterned Samples

**Figure 2:**
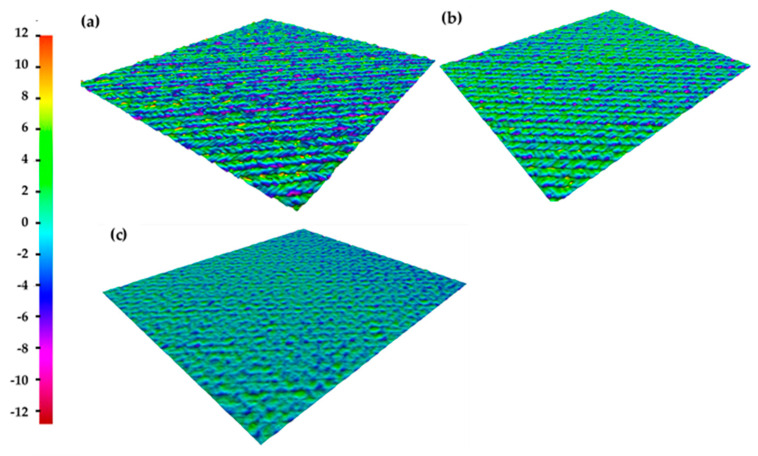
Laser patterned samples: (**a**) sample A, (**b**) sample B and (**c**) sample C.

The structure with wavelets in a regular grid was the result of laser patterning for each of the processing parameters listed above. In addition, the applied nanosecond (120 ns) pulses led to localized melting and evaporation on the surface of the Ti6Al4V alloy. On the other hand, the samples were not damaged excessive heat. Thus, no cracks or other thermal defects were observed on the surface. The surface topography for all structures is represented through the altitude map in Figure 2.

The average topography roughness is shown in Table 4. In this table, the results of surface roughness in longitudinal (long.) and transverse (trans.) according to movement of the laser beam are presented. The values were statistically analyzed by the ANOVA method with P-values 0.05. All values were statistically significant.

The topography with the highest surface roughness (structure A) had an average roughness parameter of Ra = 2.32 μm in the longitudinal direction (the direction parallel to the last path of laser beam patterning) and Ra = 2.79 μm in the transverse direction (the direction perpendicular to the last path of laser beam patterning). Topography B had an average roughness parameter of Ra = 1.93 μm in the longitudinal direction and Ra = 2.1 μm in the transverse direction. Topography C had the lowest surface roughness. This topography had an average roughness parameter of Ra = 1.4 μm in both directions. The roughness values were averaged of ten adjacent lines. The experiment proved that the average roughness in both directions of the laser structures is dependent on input laser fluence. More laser power leads to greater melting of the material. However, it should be noted that topography C was created with a fluence of 4.9 J/cm^2^, but the surface was structured four times.

Two more parameters Sku and Ssk according to ISO 25178 were measured to characterize the surface area roughness. According to [34], Sku and Ssk values can predict the surface friction properties. Parameter Sku (kurtosis) value is a measure of the sharpness of the roughness profile [34]. In comparison of laser patterns, structure C had Sku value higher than 3, which indicated that a height distribution was spiked. On the other hand, structures A and B had Sku value lower than 3, which means that a height distribution was skewed above the mean plane. Parameter Ssk (skewness) represent the degree of asperity of the roughness shape [34]. For B and C, the values were lower than zero, which indicated, that height distribution was skewed above the mean plane. On the other hand, structure A had the Ssk value higher than 0, which means that a height distribution was skewed below the mean plane.

**Figure 3:**
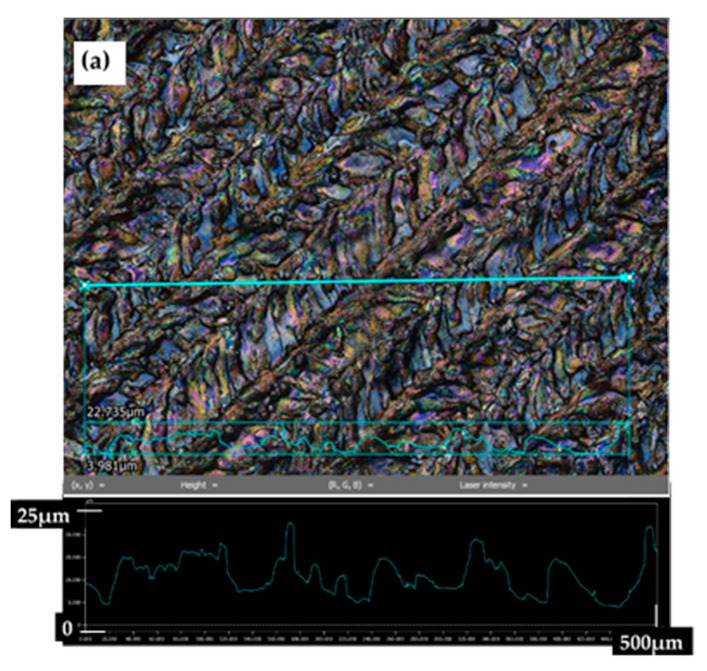

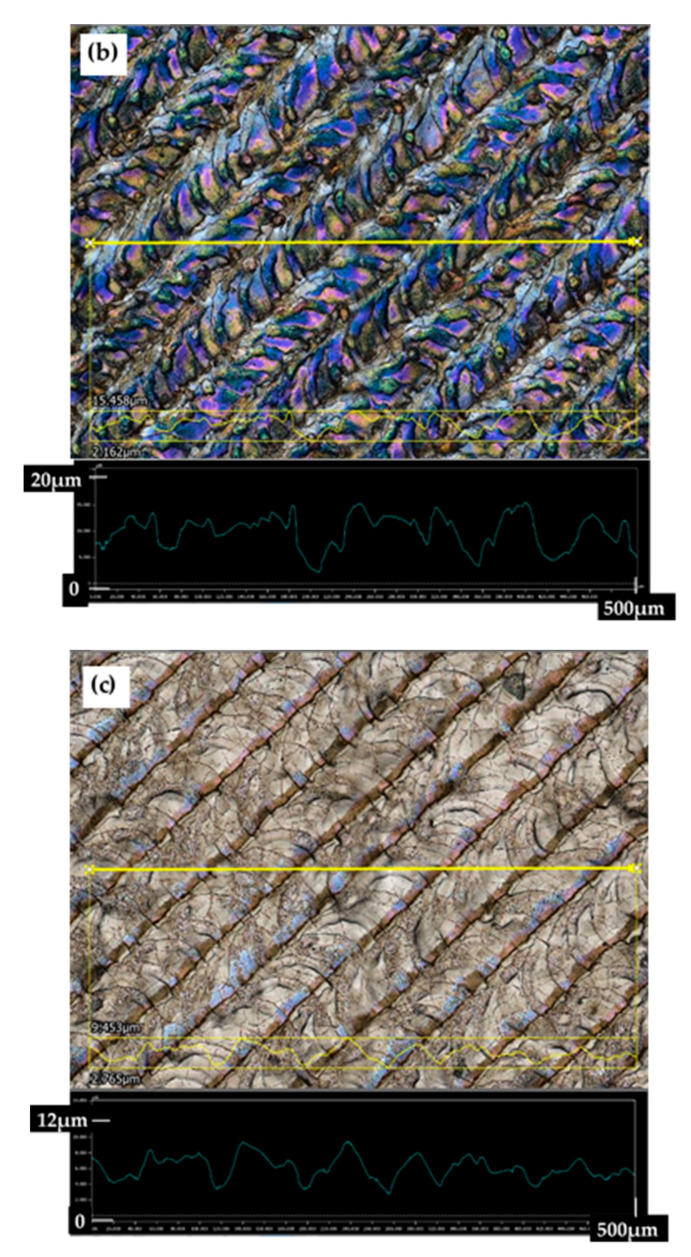
Cross-sections of the laser patterns: (**a**) structure A, (**b**) structure B, (**c**) structure C.

Another surface analysis is in Figure 3 where are the cross-sections profiles of the laser patterns. In this figure, it can be seen that the highest melting and re-solidification were observed for sample A. In addition, this sample showed the highest profile irregularity.

According to [35], an increase in surface roughness helps improve cell adhesion, migration and proliferation, which is necessary for biomaterial interaction with the human body. When the focus is on wettability, surface roughness significantly affects surface wettability. On the one hand, a higher surface roughness can lead to trapping of air in the roughness asperities and thus cause hydrophobic behavior. On the other hand, it can promote hydrophilic spreading of the droplet on the surface [36].

### 3.2. Wettability of Processed Structures

**Figure 4:**
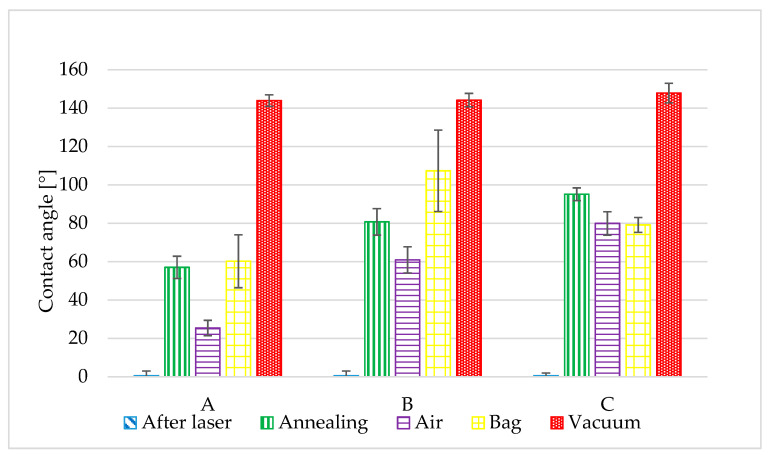
The contact angles after laser processing and at the end of the measurement period for three different laser structures A, B, C.

The laser patterned samples were highly hydrophilic immediately after the laser process. The apparent contact angle was almost zero because the droplet spread very quickly over the surface. After the laser process the surface roughness helped improve droplet spreading. Over time, more air is trapped in the roughness asperities, making the surface more hydrophobic [37]. The best hydrophilic results were obtained for structure A. This structure showed slight growth during the entire test period and after 55 days it was highly hydrophilic with a CA of 22°. On the other hand, the most hydrophobic surface was obtained by post-processing in a vacuum. This sample became hydrophobic immediately after removal from the chamber and retained its properties during the entire test period. After 55 measurement days, the vacuum sample was highly hydrophobic with a CA of around 150° for all structures. The other samples, namely the annealed sample and the sample from the polyethylene bag, remained neutral with contact angles of around 90°. All contact angles after laser processing and at the end of the measurement period (after 55 days) are shown in Figure 4.

**Figure 5:**
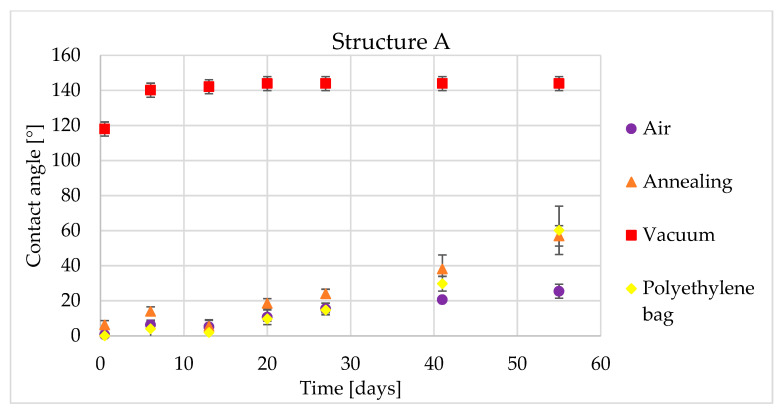
Time dependency of static contact angle for structure A and four types of post-processing.

The figures below show the time dependency of the contact angle. The error bars in these figures were obtained from three measurements on the same structure. Time dependency of CA for structure A is shown in Figure 5. 

Observed contact angles for five and 55 days are in Table 5. In this table can be clearly seen an effect of the vacuum post-processing on the creation of the hydrophobic state.

The sample which was stored in ambient air exhibited typical time behavior of laser structured samples. In the beginning, laser samples are highly hydrophilic but after a few days the samples become more hydrophobic. However, in our case, this sample stayed hydrophilic during the entire test. At the end of our test, the air-conditioned samples still exhibited hydrophilic behavior with a contact angle (CA) of approximately 80° for structure C, a CA of 22° for structure A and a CA of 61° for structure B. The best hydrophilic result was obtained for structure A. This structure showed slight growth during the entire test period and after 55 days it was highly hydrophilic with a CA of 22°.

The behavior of the samples after post-processing is more interesting. The sample stored under high vacuum conditions for 16 h exhibited hydrophobic behavior immediately after the vacuum process. All of the laser structures on this sample reached a CA of more than 120°. Moreover, sample C behaved with a CA of 143°. After another six days in ambient conditions, the CA of all patterns increased to 140°. In subsequent time measurements up to the final one, which was after 55 days, the contact angle did not change significantly, up to a maximum of five degrees.

**Figure 6:**
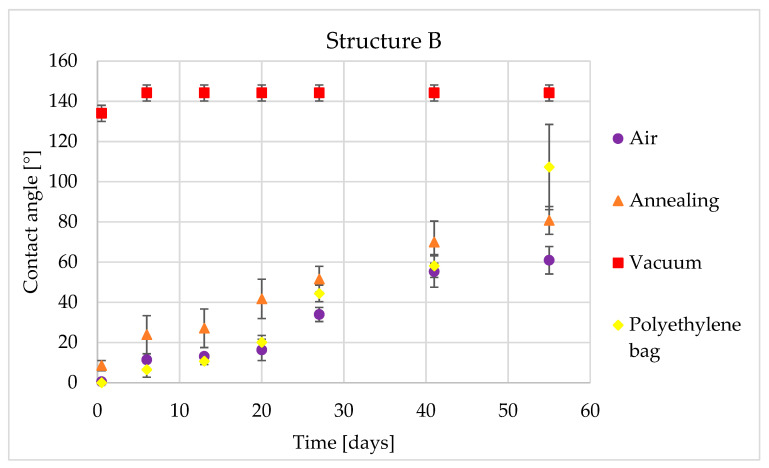
Time dependency of static contact angle for structure B and four types of post-processing.

Storage in a polyethylene bag had no significant effect on the hydrophobic transition. For patterns A and B, growth is similar to the reference sample. After 55 days of contact angle measurement, the sample stored in a polyethylene bag had a CA = 60.2° for pattern A and a CA = 79.1° for pattern C. The growth into a hydrophobic state was measured only for pattern B, where the contact angle reached 107.3° after 55 days (Figure 6).

In Table 6, there are observed contact angles for five and 55 days for structure B. In this table can be clearly seen an effect of the vacuum post-processing on the creation of the hydrophobic state and the similarity between other post-processing methods.

**Figure 7:**
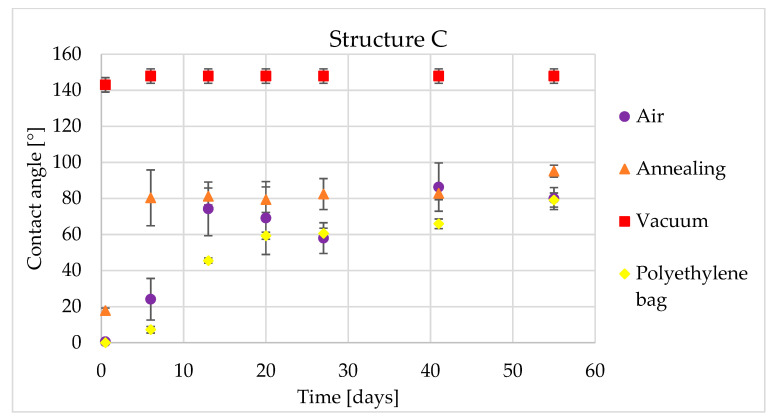
Time dependency of static contact angle for structure C and four types of post-processing.

The low temperature annealed sample behaved similarly to the sample stored in ambient air and to the polyethylene bag sample. For pattern A, the contact angle was practically identical to the polyethylene bag stored sample. The most interesting behavior was measured for pattern C (Figure 7). This pattern showed a step change in the contact angle between the first and sixth measuring day, from 17.8° to 80.3°. Then, the contact angle remained constant until the end of the experiment.

In Table 7, the contact angles for five and 55 days for structure B are shown. In this table can be clearly seen an effect of the vacuum post-processing on the creation of the hydrophobic state, as well as the very hydrophilic behavior of the air stored sample after 55 days.

A rapid transition from hydrophilic to hydrophobic was not measured for the other post-processing. Only the vacuum stored and reference samples had significant hydrophobic or hydrophilic properties respectively. As a result, only the reference and vacuum stored samples were further studied.

### 3.3. The Cleaning Processes

**Figure 8:**
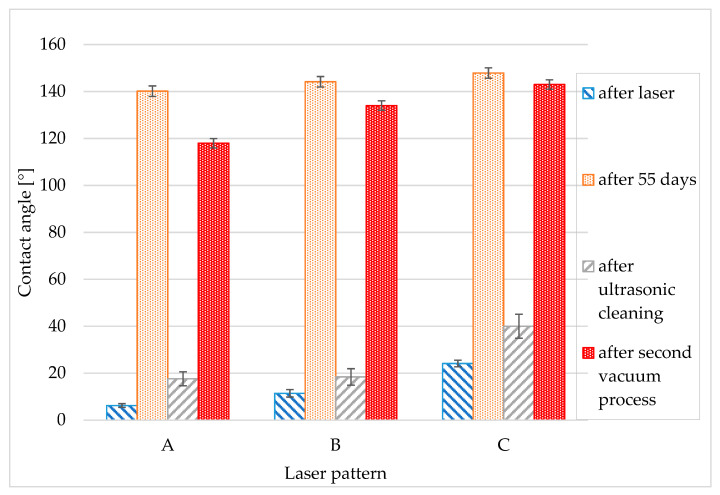
The quick transition between hydrophilic and hydrophobic states after the vacuum process and ultrasonic cleaning for three different laser structures A, B, C.

The process of ultrasonic cleaning was used after 55 days of contact angle measurement for the reference and the vacuum post-process samples. Deionized water was used to clean the samples for 15 min in an ultrasonic bath. After cleaning, the samples were dried and then the contact angles were measured. The results of this experiment showed that all of samples became hydrophilic. Figure 8 shows the contact angles after 55 days of measurement compared to the angles after ultrasonic cleaning. All the post-process effects were eliminated. Ultrasonic cleaning caused the rapid transition from hydrophobic to hydrophilic.

To prove the change from hydrophilic to hydrophobic, the vacuum process was employed again. The first sample was treated with the same high vacuum condition as in the first experiment for 12 h.

Figure 8 shows a comparison of the vacuum stored sample in the four phases of the experiment. The first bar represents the contact angle of the laser process sample (the value is not taken immediately after the laser process because the contact angle is zero, so we used the CA of the reference sample after six days), the second is the contact angle 55 days after the first vacuum post-process experiment. The third bar is the angle obtained after ultrasonic cleaning and the last bar shows the contact angle immediately after the second vacuum process. Obtained results for vacuum post-process related to Wenzel state [27].

### 3.4. Chemical Analysis of Structures

To understand the transition between hydrophilic and hydrophobic states, the surface chemistry was analyzed. According to Jagdheesh et al. [37], during high vacuum storage conditions there are carbon dissociation processes which lead to hydrophobic behavior. EDS spectroscopy was employed to confirm this theory. In this research, both hydrophilic and hydrophobic behavior was obtained for the same structure. Therefore, the surface roughness had a lower impact on wettability than the chemistry.

In the original material the chemical weight percentage amount is based on a chemical formula. This means that the amount of element for Ti6Al4V is—Al (5.5–6.75%), V (3.5–4.5%) and the rest is Ti. The rest may consist of carbon, oxygen or ferrum, in a maximum amount of 0.4% at the original surface.

**Figure 9:**
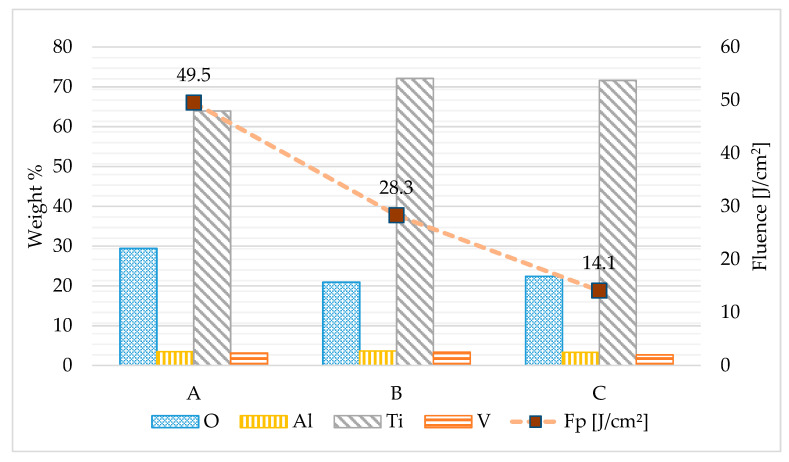
Weight % of individual elements according to fluence for laser structures A, B, C.

The relatively long nanosecond laser pulses used in this experiment influenced the melting process. EDS analysis confirmed this fact. The amount of oxides rose to 30 weight percentage for structure A. The lowest amount of oxides was found in structure C. The distribution of the other elements after laser processing is shown in Figure 9. In comparison with the original surface, the amount of aluminum decreased. This may be due to the formation of aluminum oxides. The same process was expected for vanadium and titanium. On the other hand, the volume of titanium grows. An amount of other elements does not change significantly with decreasing fluence.

Depending on the amount of carbon, the vacuum post-processed sample had a higher carbon content than the ambient air stored sample. This caused the difference between the contact angles of these two samples. Table 8 shows the weight percentage of elements for the vacuum treated sample and the air sample. For structures A and B, a slight increase in carbon content led to a change in wettability from hydrophilic to hydrophobic. There was no significant change in the carbon content of structure C, so that the contact angle did not change. Other elements presented on the surface did not change significantly. Thus, EDS analysis confirmed that chemical properties can be more important than topography in wettability studies. The most important is the carbon content, which determines whether the surface is hydrophilic or hydrophobic.

**Figure 10:**
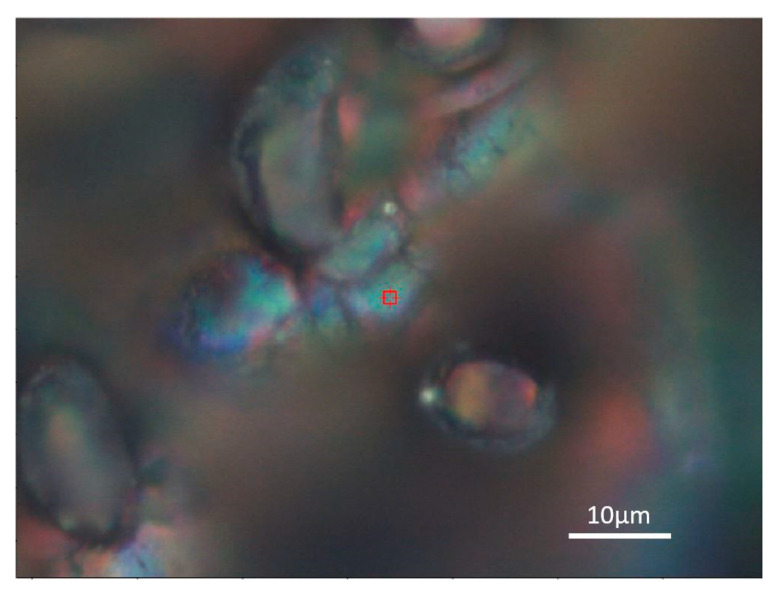
Detailed optical image of the blue-green area of the laser patterned sample.

**Figure 11:**
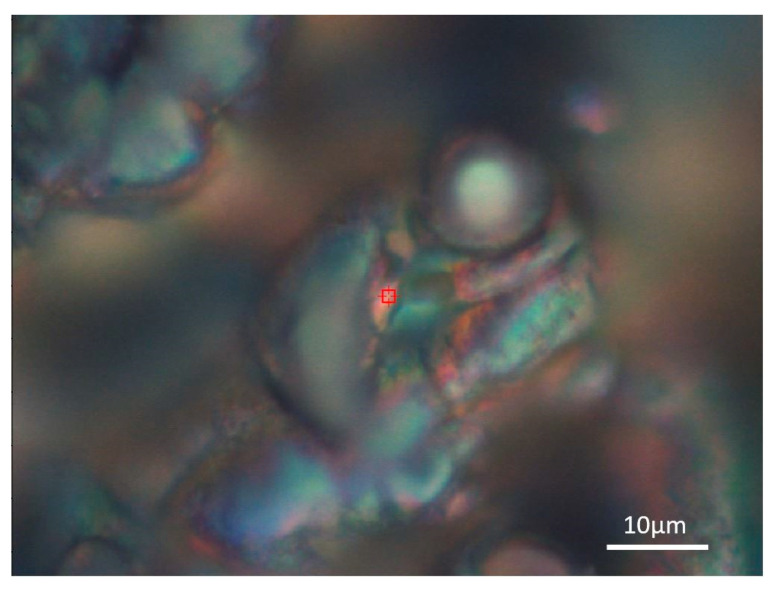
Detailed optical image of the red area of the laser patterned sample.

When the structures were studied in detail, color changes were found. For this reason, Raman spectroscopy was used to analyses chemical bonds that can detect the form of titanium oxide, especially in color areas. These color areas are shown as blue-green (Figure 10) and red (Figure 11).

**Figure 12:**
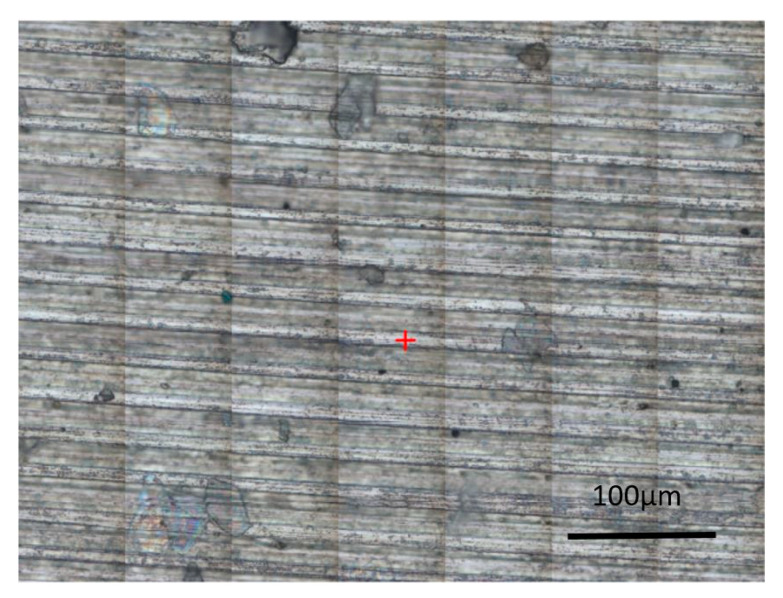
Optical image of the original structure area.

**Figure 13:**
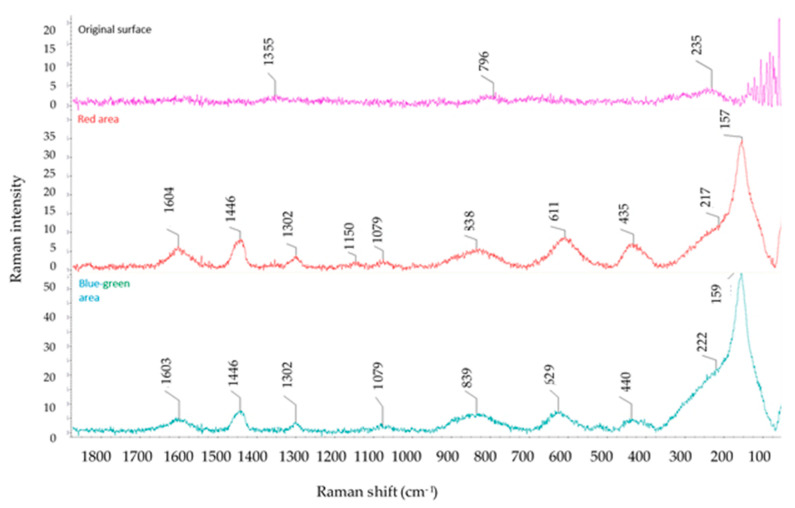
Raman intensity spectra for the original surface, red area and blue-green area.

The result of Raman spectroscopy was that both the red and blue-green areas contain titanium dioxide (TiO_2_) in anatase and rutile forms. Moreover, the blue-green areas showed a greater amount of anatase. The typical Raman shift for anatase is around 157–159 cm^−1^. A comparison of the Raman spectra for the blue-green area, red area, and the original structure (Figure 12) is given in Figure 13.

From the literature [37], we know that anatase and rutile have a positive biocompatibility effect, so their presence is highly desirable.

## 4. Discussion

In this paper, we present fast laser surface structuring with added post treatment methods to obtain both hydrophilic and hydrophobic states.

The main points for discussion are:

- The use of nanosecond pulses and the scanning strategy, led to the dominant melting process creating a micro wavelet in the regular grid. The EDS results confirmed that laser patterning leads to high surface oxidation. This phenomenon follows known information about nanosecond laser ablation [38]. Dominant effects are rapid heating in localized area to the melting point followed by vaporization. Rapid heating also caused the melt ejection to the surrounding area, forming an irregularity on the surface [38].
- Different storage condition after laser processing led to the different wettability in time. Immediately after laser process, all samples were very hydrophilic. This phenomenon is caused by removing of OH radicals from surface. Further passivation of this radicals caused hydrophobic behavior [25,39].
- In comparison of post-process methods, the vacuum process is the most suitable for production of hydrophobic surfaces. The other post - processes shows very similar behavior with slight increase of CA during time. This idea follows measurement results from EDS, where the higher volume of carbon was detected on the sample stored under vacuum conditions. This result is in accordance with [25].
- The Sku value for sample C indicates a spiked height distribution. Looking back to the nature, some functional hydrophobic surfaces such as lotus leaf or Morpho butterflies carry spike shapes [40]. The same idea was confirmed for our sample C, where the highest contact angle was observed regardless of the post-processing method.
- In a detailed view of the molten surface some color change was found, especially blue-green and red. The Raman spectrum showed that the titanium oxides found on the surface are in anatase and rutile forms. Following [41], a layer of titanium dioxide (TiO_2_) improves corrosion resistance, biocompatibility, and photocatalytic activity. In a comparison of titanium dioxide forms, an anatase improves implant properties, e.g., cytocompatibility and antibacterial properties. On the other hand, rutile phase of TiO_2_ finds application in optoelectronic devices [41,42].
- An ultrasonic cleaning process was used to test the effect of the cleaning on the wettability of the post-processed samples. As a result, all the obtained hydrophobic properties were annulled, and the samples behaved hydrophilic once again. Possible explanation of this effect could be in weak bonds between hydrophobicity caused OH radicals and surface. As reported by Li et al. [39], XPS analysis of nanosecond laser structured samples after 20 days shows an increase of carbon content from 47.5% to 54.4%, resulting in an increase of CA from 10° to 120°.
- Hydrophobic behavior was achieved again by re-applying a highly vacuum process on the same sample. This effect was caused by redeposition of an OH radicals to the surface as described by Jagdheesh et. al. [25].
- It was shown that the change in wettability was mainly influenced by surface chemistry regardless of the surface roughness and the microstructure. For further research, this brings an idea of changing surface topography by laser machining together followed by applying a low surface energy coating. Some recent studies confirm this idea for enamel coatings on inorganic glass [43] or hydroxyapatite coating applied on Ca-P ceramics for medical implants [44].

## 5. Conclusions

A repeatable process of rapid wettability change for various nanosecond laser patterns has been demonstrated. The process includes different further processing methods.

In addition, laser surface texturing with nanosecond laser is fast method for modification of surface topography. This thermal process also leads to surface melting and, especially for titanium alloy, forming TiO_2_ in the form of anatase and rutile. Both the forms were indicated on the laser textured surface, where they can play a significant role according to the specific surface properties. Surface topography was characterized by Ssk and Sku values. These characteristics can be used for predicting surface wettability according to our results. For sample with the lowest surface roughness, Sku value indicated spiked height distribution. This behavior was observed with the highest tendency to hydrophobicity.

Four different post-processing methods were studied: (i) high vacuum, (ii) low temperature annealing, (iii) PE bag storing, and (iv) air condition storing. Different storage condition after laser processing led to the different wettability in time. Immediately after laser process, all samples were very hydrophilic. High vacuum processing gave hydrophobic properties of the surface. According to EDS analysis, it was caused by a higher amount of carbon on the surface.

The values of contact angle (CA) indicated a hydrophobic state for almost all process at the end of the measuring period. Nevertheless, after ultrasonic cleaning, the surfaces became hydrophilic again. A possible explanation for this fact is in the very weak bonds between hydrophobic hydroxide (OH) radicals.

The obtained results can be applied for dental implants, where the surface topography and wettability play a significant role. The next steps should lead to the confirmation of the observed phenomena in this field. Another research goal is in a combination of laser surface texturing together with applying a low surface energy coating. This approach is suitable for preparing long-term super-hydrophobic surfaces.

## Figures and Tables

**Table 1:** Chemical composition of Ti6Al4V rollers.

Element	N	C	H	Fe	O	Al	V	Ti
Weight %	0.006	0.02	0.001	0.16	0.166	6.36	4.2	89.087

**Table 2:** Laser parameters used to create structures.

Indication	Power [W]	Frequency [kHz]	Scan Speed [mm/s]	Number of Repetitions	Pulse Overlap [%]	Intensity [J/cm^2^]	Pulse Energy [mJ]
A	35	4	120	1	80	49.5	8.75
B	20	4	120	1	80	28.3	5.00
C	10	4	120	4	80	14.1	2.5

**Table 3:** Indication of samples and post-processing.

Sample number	Type of Post-Processing	Storage Time
Sample no. 1	Vacuum	16 h (8·10^−7^ Pa)
Sample no. 2	Low temperature annealing	8 h (100 °C)
Sample no. 3	Ambient air	Entire test period
Sample no. 4	Polyethylene (C_2_H_4_) bag	Entire test period

**Table 4:** Surface roughness parameters of the laser patterns.

Roughness	Laser Pattern						Original Surface
	Structure A		Structure B		Structure C		
	Long.	Trans.	Long.	Trans.	Long.	Trans.	
Ra [µm]	2.32 ± 0.13	2.79 ± 0.04	1.93 ± 0.29	2.10 ± 0.04	1.40 ± 0.24	1.45 ± 0.21	0.32
Rz [µm]	17.70 ± 0.11	20.51 ± 0.5	16.99 ± 2.52	17.65± 1.69	14.60 ± 3.57	14.82 ± 4.19	1.70
Sa [µm]	2.90 ± 0.06		2.47 ± 0.05		1.56 ± 0.18		
Sz [µm]	32.21 ± 1.37		41.59 ± 0.61		50.71 ± 6.54		
Sku [μm]	2.73 ± 0.04		2.71 ± 0.12		3.14 ± 0.31		
Ssk [μm]	0.24 ± 0.02		−0.11 ± 0.001		−0.02 ± 0.008		

**Table 5:** Comparison of droplet images for structure A after 5 and 55 days.

Number of Days after Laser Process	Air	Annealing	Vacuum	PE Bag
5 days	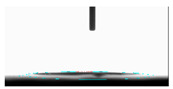	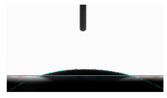	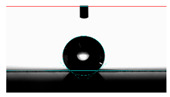	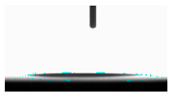
55 days	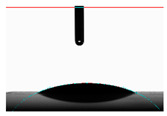	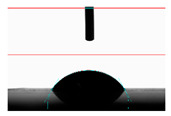	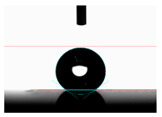	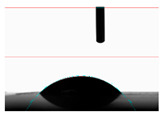

**Table 6:** Comparison of a droplet images for structure B after 5 and 55 days.

Number of Days after Laser Process	Air	Annealing	Vacuum	PE Bag
5 days	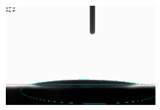	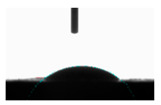	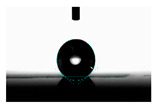	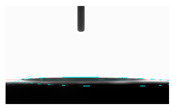
55 days	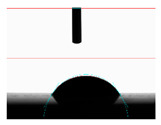	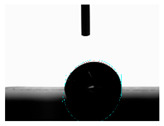	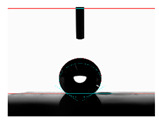	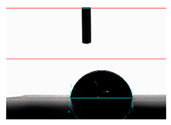

**Table 7:** Comparison of a droplet images for structure C after 5 and 55 days.

Number of Days after Laser Process	Air	Annealing	Vacuum	PE Bag
5 days	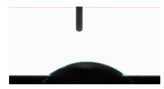	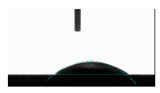	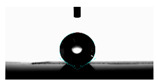	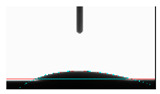
55 days	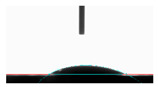	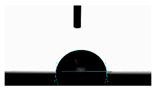	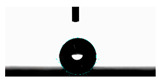	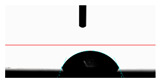

**Table 8:** Weight % of elements in the vacuum process sample and air stored samples.

Weight %	Post-Process	C	O	Al	Ti	V
A	air	1.99	28.29	3.45	63.16	3.11
	vacuum	3.52	28.74	3.28	61.65	2.82
B	air	1.51	20.14	3.64	71.42	3.29
	vacuum	4.41	21.92	3.49	66.66	3.51
C	air	3.2	21.15	3.23	69.83	2.6
	vacuum	3.59	18.85	3.21	71.88	2.47

## Data Availability

Data is contained within the article.
